# Current pool of ultimate collection of mitochondrial DNA from remnants of Kalash

**DOI:** 10.1080/23802359.2021.1952119

**Published:** 2021-07-15

**Authors:** Muhammad Hassan Siddiqi, Allah Rakha, Khushbukhat Khan, Tanveer Akhtar

**Affiliations:** a Department of Zoology, University of the Punjab, Lahore, Pakistan; b Department of Zoology, Government College Women University, Sialkot, Pakistan; c Department of Forensic Sciences, University of Health Sciences, Lahore, Pakistan; d Atta-ur-Rahman School of Applied Biosciences (ASAB), National University of Sciences and Technology (NUST), Islamabad, Pakistan

**Keywords:** Mitochondrial DNA, mtDNA control region, hypervariable segments, Kalash population, Pakistan

## Abstract

The mitochondrial DNA (mtDNA) complete control region coverage of 111 individuals from Kalash population of Pakistan has been presented for forensic applications and to infer their genetic parameters. We detected in total 14 different haplotypes with only five unique and nine shared by more than one individual. This population has come up with quite lower haplotype diversity (0.8393) and very higher random match probability (0.1682), and ultimately lower power of discrimination (0.832). Additionally, haplogroup distribution reveals the genetic ancestry of Kalash, mainly from West Eurasia (98.8%) and very little from South Asia (0.9%). Neither African lineages nor East Asian genetic segments were detected among these Kalash. This study will contribute to the database development for forensic applications as well as to track the evolutionary highlights of this ethnic group.

## 1. Introduction

Pakistan is assumed to be on the crossroad of modern humans out of Africa and it is one of first terrain where contemporary humans inhabited. On the basis of culture and language, Pakistan is usually divided into 16 ethnic groups of miscellaneous pedigrees. The major ethnic groups include the Punjabis, Pathans, Sindhi, Saraiki, Muhajir, Balochi, Kalashi, and Makrani (Rakha et al. 2011). The Kalasha or Kalash people are a group of Indo-European Indo-Iranian speaking people living in the Chitral district of Khyber-Pakhtunkhwa province of Pakistan (Denker 1981; Ayub et al. 2015). This unique tribe amongst Indo-Aryan peoples of Pakistan comes from a Dardic family. Census’ outcome reports its population size to be 5000 individuals which shows its religious minority accompanied by rich cultural attributes (Ayub et al. 2015). Generally, the Kalash people by dint of their legends and mythos are associated to ancient Greece, but traditionally they are much nearer to Vedic and pre-Zoroastrians (Mela-Athanasopoulou, 2011) . It is proudly claimed by some of Kalash people of being descendants of Alexander the Great’s soldiers but wide-ranging genetic studies do not support this claim (Williams et al. 2015). Autosomal and Y-chromosome short tandem repeat (STR) analysis suggested no admixture of Greek genetic element in gene pool of Kalash population (Mansoor et al., 2004; Firasat et al., 2007) . Mitochondrial DNA (mtDNA) analysis on Kalash population also does not provide much insight on their evolutionary history because of low cohort size (44 individuals) studied in them (Quintana-Murci et al., 2004). Further, in contrast to previous mtDNA-based Kalash studies where haplogroup assignment is done on the basis of high-resolution RFLP analysis (Ayub et al. 2015), in this study Kalash characterization on the basis of maternal inheritance is done by sequencing ∼1122 bp long entire control region of mtDNA. So, this study with the highest number of Kalash samples (111) so far is aimed to analyze the mtDNA control region of the genome to identify the haplogroup composition of Kalash.

## 2. Materials and methods

### 2.1. DNA extraction, amplification, and sequencing

**Figure 1.**
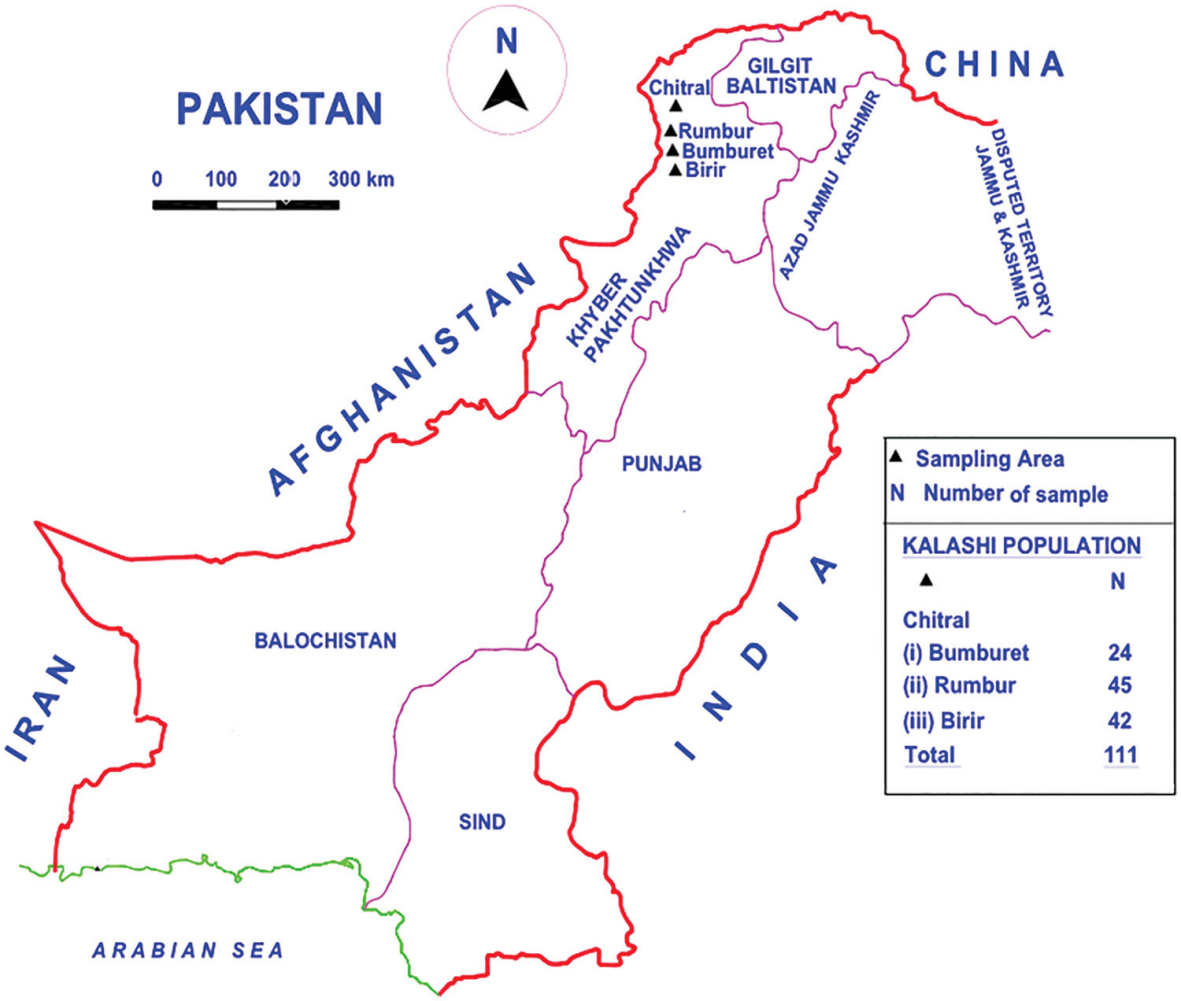
Map of Pakistan showing study area for Kalash population.

The study was approved from the ethical committee of Department of Zoology, University of the Punjab, Lahore, Pakistan. Blood samples of 111 healthy and maternally unrelated Kalash people were collected with oral and written consent of participants (Figure 1). Further, individuals living in the area for three generations and were between the age of 18–70 years were sampled. DNA was extracted from preserved blood samples using DNA extraction QIAamp^®^ DNA Mini kit (Qiagen, Hilden, Germany). DNA quantitation was done using NanoDrop 1000 spectrophotometer (Thermo Scientific, Wilmington, DE). After the quantification of the genomic DNA, the mtDNA complete control region was amplified by the same condition as described in (Siddiqi et al. 2015). Sequencing of the entire mtDNA control region spanning nucleotide positions 16,024–16,569 and 1–576 was done using Big Dye Terminator Cycle Sequencing version 3.1 Ready Reaction Kit (Applied Biosystems, Carlsbad, CA) according to the manufacturer’s instructions.

### 2.2. Data analysis

All samples were sequenced bi-directionally and evaluated twice as recommended by ISFG (Parson et al. 2014). Applications of online available tools like MitoTool (Fan and Yao 2011), mtDNA profiler (Yang et al. 2013), and HaploGrep (Weissensteiner et al. 2016), making use of PhyloTree as classification tree, were used to evaluate the quality of mtDNA data. Haplogroup assignment was done using the most updated PhyloTree build version 17 (Utrecht, The Netherlands (Van Oven and Kayser 2009). The population statistical parameters haplotype diversity, random match probability, and power of discrimination were statistically calculated by using DnaSP version 6 (Rozas et al., 2017). Analysis of molecular variance (AMOVA) and pairwise *F*_ST_ values was calculated using Arlequin software version 3.5 (Excoffier and Lischer 2010, Institute of Ecology and Evolution, University of Bern, Switzerland). The Kalash data were compared with the samples from other ethnic groups from Pakistan (Pathan (Rakha et al. 2011), Kashmiri (Rakha et al. 2016), Saraiki (Hayat et al. 2015), and Makrani (Siddiqi et al. 2015), ) and with population datasets from other countries including Uzbekistan, China, Dubai, Egypt, Iraq, Kuwait, Laos, Thailand, and Vietnam (Alshamali et al. 2008; Irwin et al. 2008, 2009; Saunier et al. 2009; Zimmermann et al. 2009; Irwin et al. 2010). Median-joining haplotype network was constructed using the software NETWORK (Kong et al., 2016) .

## 3. Results and discussion

In this study, we present the data of 111 Kalash people for mtDNA control region. Mitochondrial complete control region sequences were submitted and available through GenBank accession (KM358270-KM358380). We observed the 14 different haplotypes (five unique and nine shared) with only 47 polymorphic sites. The detected haplotypes, their respective frequencies, and haplogroups have been presented in Table 1. The Kalash population has come up with mtDNA genetic diversity 0.8393, random match probability 0.1682, and power of discrimination 0.832 as shown in Table 2. Total of eight haplogroups were found for Kalash population. Among them, the highest frequency was observed for haplogroup R0a’b (28.8%) in Kalash population (this study) and no evidence was found in Makrani (Siddiqi et al. 2015), Pathans (Rakha et al. 2011), Hazara (Rakha et al. 2017), and Saraiki (Hayat et al. 2015) for this haplogroup.

**Table 1.** The observed haplotypes, haplogroups, their frequencies, and continental origin in Kalash population from Pakistan.

Haplotype ID	Frequency (%)	Differences to the rCRS (309ins, 315ins, 524ins, and 16,519 were disregarded)	Haplogroup	Haplogroup origin
h1	1 (0.9)	16354T, 199 C,263G	H2a1	WEA
h2	2 (1.8)	16354T, 263 G	H2a1	WEA
h3	1 (0.9)	16354T,263G	H2a1	WEA
h4	7 (6.3)	16356C, ,73 G,195C,198T,263G,499A	U4	WEA
h5	31 (27.9)	16362C, 58 C,60.1T,64T,263G	R0a + 60.1T	WEA
h6	1 (0.9)	16240G,16362C ,58 C,60.1T,64T,263G	R0a + 60.1T	WEA
h7	1 (0.9)	16223T,16289G, 73 G,263G,489C,511T	M65a + @16311	SA
h8	1 (0.9)	16223T,55.1T,57C,59C,62T,73G,146C,152C,195C,263G,489C	?	
h9	24 (21.6)	16134T,16356C ,73 G,152C,195C,263G,499A	U4a1	WEA
h10	2 (1.8)	16071T ,16527 T,73G,152C,263G	R2	WEA
h11	5 (4.5)	16071T,16111T,16147T,16203G,16311C,73G,150T,263G	R2	
h12	16 (14.4)	16069T,16126C,16193T,16274A,16278T,73G,150T,152C,263G,295T,489C	J2b1a	WEA
h13	12 (10.8)	16051G,16129C,16154C,16248T,16362C,16391A,73G,152C,217C,263G,340T,508G	U2e1h	WEA
h14	7 (6.3)	16051G,16129C,16154C,16248T,16362C,73G,152C,217C,263G,340T,508G	U2e1h	WEA

rCRS: revised Cambridge reference sequence; WEA: West Eurasian; SA: South Asian

**Table 2.** Statistical parameters for different populations from Pakistan.

Parameter	Kalash	Makrani (Siddiqi et al. 2015)	Saraiki (Hayat et al. 2015)	Pathan (Rakha et al. 2011)	Kashmiri (Rakha et al. 2016)	Sindhi (Yasmin et al. 2017)	Hazara (Rakha et al. 2017)	Balti (Khan et al. 2020)	Chitral (Rehman 2021)
Total no. of samples	111	100	85	230	317	88	319	52	10
Haplotypes	14	70	63	193	251	66	189	43	10
Unique haplotypes	5	54	58	128	201	50	124	37	10
Polymorphic positions	47	142	140	215	230	170	217	92	–
Random match probability	0.1682	0.0408	0.0542	0.0065	0.0054	0.0188	0.0085	0.025	–
Power of discrimination	0.832	0.9592	0.9458	0.8348	0.7918	0.9811	0.9915	0.9748	–
Genetic diversity	0.8393	0.9688	0.9570	0.993	0.9977	0.9924	0.9945	0.9939	0.9589

**Figure 2.**
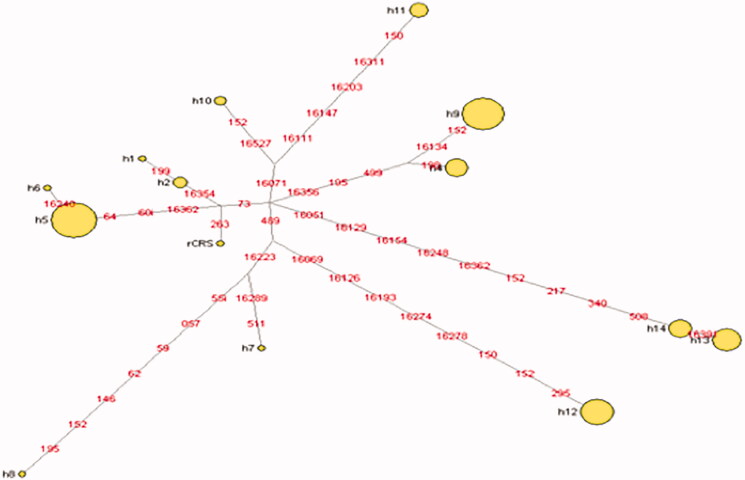
Median-joining haplotype network of the Kalashi population (14 haplotypes) Mutations 309.1 C, 309.1CC, 315.1 C, 16,182 C, 16,183 C, and 16,519 C, as well as length variation in the AC stretch spanning pos. 515–524, were ignored for network construction. Circle sizes are proportional to the number of mtDNAs with that haplotype and branch lengths are proportional to nucleotide changes.

The genetic diversity of Kalash is exceptionally low, reflecting their conserved traditions in every aspect of life. In another recent study, Kalash has been demonstrated showing the largest genetic distance with respect to other ethnic groups. Moreover, Kalash has been reported as genetic outlier who does not fall in any of definite genetic identity ( Rosenberg et al., 2002). This could be due to the strong impact of evolutionary force like genetic drift causing reduction from prior large population to current smaller population. Likewise, being in sub-continent, Kalash has no visible traces of east or south Asian genetic lineages which could be due to a founder event where fewer numbers of individuals isolated and overtime thrived into present population. The findings of this study are parallel with (Cardoso et al. 2012) study outcomes on Waorani tribe from Amazon. The Kalash with only five unique haplotypes depicting them as conserved population with poor gene pool and lower haplotype diversity mainly due to higher endogamy practices. Very few populations are reported with such a less mtDNA haplotype diversity. Only 11 unique haplotypes were reported in Kichwa population from Ecuador ( Baeta et al., 2012) while only three unique haplotypes were reported in Waorani tribe from Ecuadorian Amazon (Cardoso et al. 2012). The Kalash dataset was compared to other 20 populations by computing AMOVA (Table S1). The majority of observed variance (96.11%) was attributable to differences within populations, and only 3.89% represented differences among populations. The *F*_ST_ pairwise differences calculated were found comparatively higher between the populations from East and South East Asia, whereas lower between South and West Asian populations (Table S2). The most dominant haplogroups in the mtDNA of Kalash population are U4, R0a, U2e, and J2 indicating the occurrence of Western Eurasian influence (Table 1). Western Eurasian origin of Kalasha people was also indicated by Rahman et al. (2020). The median-joining network analyses of Kalash population have shown a considerable divergence between haplotypes. This network shows many independent branches giving rise to many sub-branches that are separated by several mutations (Figure 2). The Kalash population is the best example to represent population complexity of the Central Asian region and had very low frequency of South Asian lineage haplogroups.

So, the Kalash population after comparison with rCRS (NC_012920) has been presented with total only 14 haplotypes (nine shared and five unique haplotypes). The haplogroup R0a + 60.1T (West Eurasian was observed most frequently 28.8% in this population affirming the previous studies as far as West Eurasian dominance is concerned. This data is in accordance with Rahman et al. (2020) study outcomes. However, the number of individuals (38 samples) included in that study was less than this study cohort size (111 samples). In this population, we observed the West Eurasian haplogroups with unmatched frequency (98.2%) including R0a + 60.1T (28.8%), U4 (27%), U2e1h (17.1%), J2b1a (14.4%), R2 (6.3%), and H2a1 (3.6%) compared with other Pakistani populations. South Asian representation is led by only one haplogroup M65a* (0.9%). The limited haplogroup diversity is most probably the consequence of the isolation of the population.

## Data Availability

Control region sequences of mtDNA of 111 individuals from Kalash population are available on accession number KM358380.1 to KM358270.1 (https://www.ncbi.nlm.nih.gov/popset/698364991).
